# The effect of statin treatment on circulating coenzyme Q10 concentrations: an updated meta-analysis of randomized controlled trials

**DOI:** 10.1186/s40001-018-0353-6

**Published:** 2018-11-10

**Authors:** Hua Qu, Yan-yan Meng, Hua Chai, Fang Liang, Jia-yi Zhang, Zhu-ye Gao, Da-zhuo Shi

**Affiliations:** 1 China Academy of Chinese Medical Sciences, Beijing, China; 2 China Heart Institute of Chinese Medicine, China Academy of Chinese Medical Sciences, Beijing, China; 3 Beijing University of Traditional Chinese Medicine, Beijing, China; 4 Xiyuan Hospital, China Academy of Chinese Medical Sciences, Beijing, China

**Keywords:** Statin treatment, Circulating CoQ10, Meta-analysis, Pharmaceutical metabolism

## Abstract

**Background:**

The effect of statin treatment on circulating coenzyme Q10 (CoQ10) has been studied in numerous randomized controlled trails (RCTs). However, whether statin treatment decreases circulating CoQ10 is still controversial.

**Methods:**

PubMed, EMBASE, and the Cochrane Library were searched to identify RCTs to investigate the effect of statin treatment on circulating CoQ10. We calculated the pooled standard mean difference (SMD) using a fixed effect model or random effect model to assess the effect of statin treatment on circulating CoQ10. The methodological quality of the studies was determined according to the Cochrane Handbook. Publication bias was evaluated by a funnel plot, the Egger regression test, and the Begg–Mazumdar correlation test.

**Results:**

Twelve RCTs with a total of 1776 participants were evaluated. Compared with placebo, statin treatment resulted in a reduction of circulating CoQ10 (SMD, − 2.12; 95% CI, − 3.40 to − 0.84; *p* = 0.001), which was not associated with the duration of statin treatment (Exp, 1.00; 95% CI, 0.97 to 1.03; *p* = 0.994). Subgroup analysis demonstrated that both lipophilic statins (SMD, − 1.91; 95% CI, − 3.62 to 0.2; *p* = 0.017) and hydrophilic statins (SMD, − 2.36; 95% CI, − 4.30 to − 0.42; *p* = 0.028) decreased circulating CoQ10, and no obvious difference was observed between the two groups (SMD, − 0.20; 95% CI, − 0.208 to 0.618; *p* = 0.320). In addition, both low-middle intensity statins (SMD, − 2.403; 95% CI, − 3.992 to − 0.813; *p* < 0.001) and high intensity statins (SMD, − 1.727; 95% CI, − 2.746 to − 0.709; *p* < 0.001) decreased circulating CoQ10. Meta-regression showed that the effect of statin on decreasing circulating CoQ10 was not closely associated with the duration of statin treatment (Exp, 1.00; 95% CI, 0.97 to 1.03; *p* = 0.994).

**Conclusions:**

Statin treatment decreased circulating CoQ10 but was not associated with the statin solution, intensity, or treatment time. The findings of this study provide a potential mechanism for statin-associated muscle symptoms (SAMS) and suggest that CoQ10 supplementation may be a promising complementary approach for SAMS.

**Electronic supplementary material:**

The online version of this article (10.1186/s40001-018-0353-6) contains supplementary material, which is available to authorized users.

## Introduction

Statins are widely used in the prevention and treatment of coronary heart disease [1]. Numerous large-scale studies have demonstrated that statins substantially reduce cardiovascular morbidity and mortality in both primary and secondary prevention, in both genders and in all age groups [2–4]. However, statin-associated muscle symptoms (SAMS), covering a broader range of muscle symptoms following statin treatment, are an important reason for statin discontinuation [5]. A previous study demonstrated that SAMS result in significantly high discontinuation rates of statin treatment (up to 75%) within 2 years of initiation [6], and in 65% of former statin users, the main reason for statin non-adherence or discontinuation was the onset of side effects, predominantly SAMS [7]. Non-adherence or discontinuation of statin treatment contributes to adverse cardiovascular outcomes. A meta-analysis showed a 15% lower cardiovascular risk in patients who adhered to statin treatment compared with those with low adherence [8]. Studying the possible mechanism and therapeutic approach of SAMS could decrease the cardiovascular risk in patients who are intolerant to statins due to SAMS [9]. The European Atherosclerosis Society has proposed four strategies for treating SAMS, including re-challenge with alterative statin therapy, lower or intermittent statin therapy, non-statin-based lipid-lowering therapy, and complementary therapies. Complementary therapies, such as coenzyme Q10 (CoQ10) supplementation, might be a promising method to manage SAMS [9]. The mechanism of SAMS is currently unclear, but changes in circulating coenzyme Q10 concentration may be involved in the pathological process. Exploring the level of circulating CoQ10 following statin treatment may explain potential mechanisms and suggest possible complementary approaches for SAMS.

CoQ10 is a naturally occurring, fat-soluble quinone located in the hydrophobic portions of cellular membranes [10], and it plays an important role in mitochondrial energy metabolism and stabilization of muscle cell membranes [11, 12]. A previous animal study demonstrated that statin treatment could lead to the reduction of circulating CoQ10 [13]. However, the findings concerning changes in circulating CoQ10 following statin therapy have been inconsistent in clinical studies. A previous meta-analysis [14] only included 6 clinical studies, and several RCTs, including statin treatment for a period of 24 weeks, have been published since, and these studies have provided new evidence. Therefore, the present meta-analysis of RCTs was designed to reassess the effect of statin treatment on circulating CoQ10.

## Methods

This study was performed according to the guidelines of the 2009 Preferred Reporting Items for Systematic Reviews and Meta-Analysis statement (PRISM) (Additional file 1: Table S1) [15].

### Data source and search strategies

Two reviewers (Hua Qu and Hua Chai) searched PubMed, EMBASE, and the Cochrane Library with no language restrictions from inception to January 2018 to identify all existing literature. Mesh terms and free-text terms were used in each database with the following relevant keywords: “statin treatment” AND “coenzyme Q10” AND “randomized controlled trials.” A manual search was also performed to identify relevant references from the selected articles and published reviews. The studies were eligible if they met the following inclusion criteria: (1) randomized, controlled, parallel, or crossover trial, (2) the intervention group received statin and the comparison group received placebo, or the intervention group received lipophilic statin and the comparison group received hydrophilic statin, and (3) the outcome regarding circulating CoQ10 (plasma CoQ10 or serum CoQ10) was available.

### Data extraction and assessment of study quality

Two reviewers (Hua Qu and Yan-yan Meng) extracted data independently. If a disagreement occurred, it was resolved by consulting with a third investigator (Da-zhuo Shi). We contacted the authors if the article was only published with an abstract, and the studies without original data were excluded. The following data were extracted from each individual eligible study: (1) the first author’s name and publication year, (2) intervention duration, (3) inclusion criteria, (4) participant number, (5) participants’ age, (6) percentage of males, and (7) clinical outcomes. The methodological quality of eligible studies was determined according to the recommendation of the Cochrane Handbook [16].

### Statistical analysis

In this meta-analysis, continuous data were used to analyze the standard mean difference (SMD) with a 95% confidence interval (CI) for the effect size. Heterogeneity in the eligible studies was evaluated using the Chi-square test based on Cochran’s *Q* test and *I*^2^ statistic at the *p* < 0.10 level of significance, and quantification of heterogeneity was calculated using the *I*^2^ metric, which describes the percentage of total variation estimated to be due to heterogeneity rather than chance. When P for the heterogeneity was < 0.1 and *I*^2^ ≥ 50%, the inter-study heterogeneity was considered statistically significant. The selection of the random or fixed effect model was based on the heterogeneity analysis. The fixed effect model was applied if *I*^2^ < 50%, and the random effect model was chosen if *I*^2^ ≥ 50%. We performed subgroup analysis and meta-regression to detect the potential sources of heterogeneity in the condition of *I*^2^ ≥ 50%. Sensitivity analysis was performed to assess the robustness of the pooled SMDs by eliminating one study at a time. The publication bias was evaluated by funnel plot, Egger regression, and the Begg–Mazumdar correlation test. Statistical analysis was performed using Stata (version 12.0). There is no registered protocol for the present meta-analysis.

## Results

### Description of included studies

**Fig. 1:**
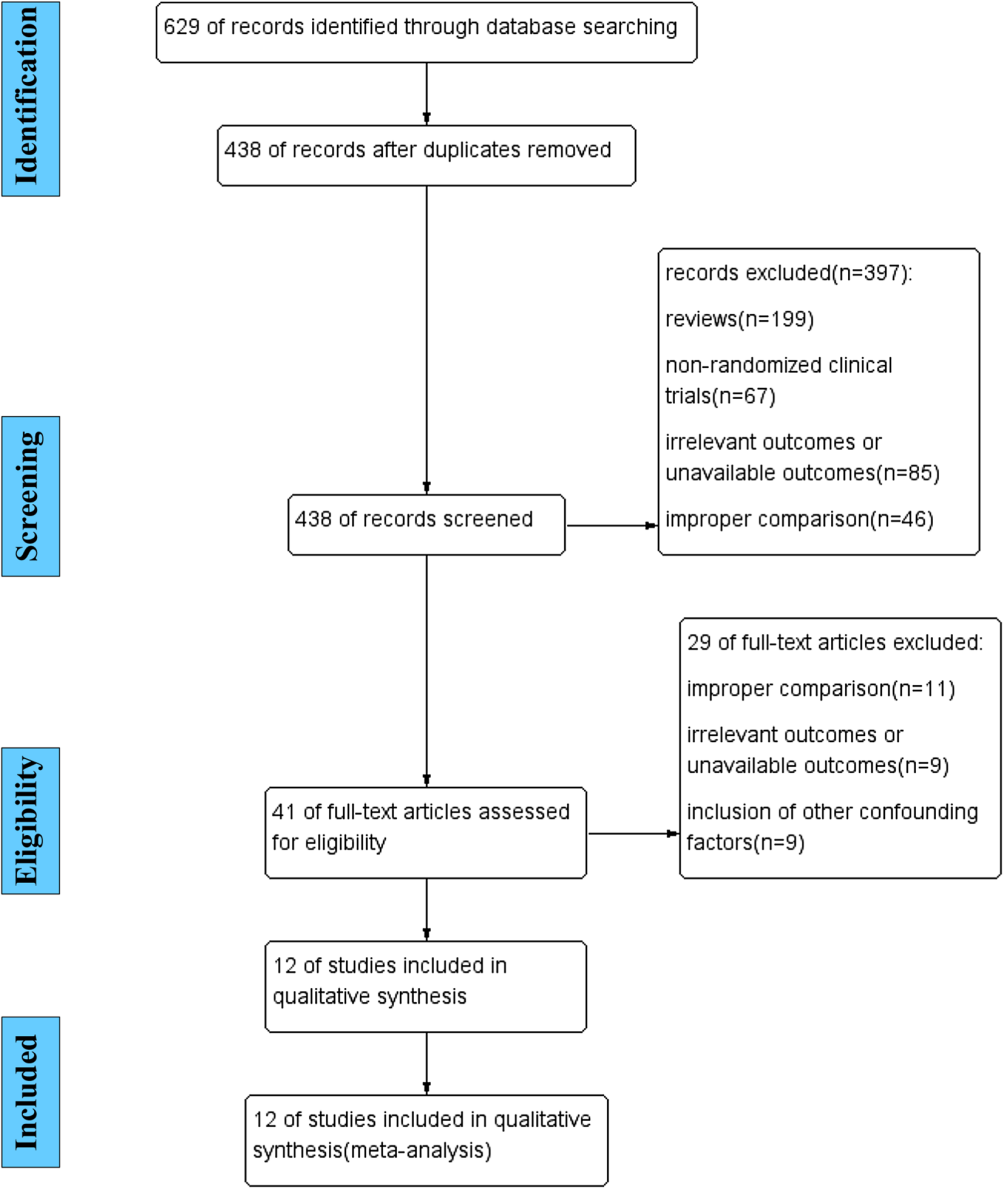
Literature search process and study selection

Six hundred and twenty-nine studies (298 from PubMed, 304 from EMBASE, and 27 from the Cochrane Library) were identified, and 191 articles were excluded as duplicated records. After the titles and abstracts of the articles were screened, 397 articles were excluded due to review format, improper study type, and/or improper comparisons. After the remaining 41 full-text articles were reviewed, 29 articles were excluded due to improper comparisons, irrelevant outcomes, and/or unavailable outcomes. Finally, 12 articles [17–28] with 1776 participants published in English from 1993 to 2018, with sample sizes ranging from 19 to 1103 participants and intervention durations ranging from 14 days to 26 weeks, were entered into our meta-analysis (Fig. 1, Table 1). Nine RCTs [17–21, 23–25], including 11 study arms (822 participants in the statin treatment group vs. 830 participants in the placebo group), evaluated the effect of statins (statins vs. placebo) on circulating CoQ10, and 4 RCTs [22, 25, 26, 28], including 7 study arms (94 participants in a lipophilic statin group vs. 115 participants in a hydrophilic statin group), evaluated the effect of different soluble statins (lipophilic statins vs. hydrophilic statins) on circulating CoQ10.

**Table 1:** Basic characteristics of the participants

Study	Duration	Inclusion criteria	Participants (T/C)	Age (years)		Male (%)		Outcome
				T	C	T	C	
Jula 2015	12 weeks	Dyslipidemia patients	120 (60/60)	48.4 ± 6.2	48.0 ± 6.2	100	100	Serum CoQ 10 concentrations
Oranje 2001	3 months	Type 2 diabetes patients	19 (9/10)	64 ± 8	63 ± 8	100	70	Plasma CoQ 10 concentrations
Päivä 2005	8 weeks	Dyslipidemia patients	48 (32/16)	31–69	31–69	N	N	Plasma CoQ 10 concentrations
Strey 2005	6 weeks	Heart failure (NYHA II or III) with LVEF < 40%	48 (24/24)	N	N	N	N	Plasma CoQ 10 concentrations
Mortensen 1997	6 weeks	Dyslipidemia patients	45 (23/22)	52.9 (32,69)	54.8 (42,67)	60.9	40.9	Serum CoQ 10 concentrations
Morrison 2017	24 weeks	Patients with HIV	147 (71/75)	45 (41,51)	47 (39,53)	81	76	Plasma CoQ 10 concentrations
McMurray 2010	3 months	Heart failure (NYHA II or III) with LVEF < 40%	1103 (551/552)	N	N	N	N	Serum CoQ 10 concentrations
Ghirlanda 1993	3 months	Dyslipidemia patients	30 (20/10)	47 ± 8; 49 ± 10	47 ± 8	60; 50	70	Plasma CoQ 10 concentrations
Chitose 2014	6 months	STEMI patients	75 (38/37)	64.1 ± 11.8	65.8 ± 12.4	73.7	75.7	Plasma CoQ 10 concentrations
Berthold 2006	14 days	Healthy subjects	48 (24/24)	31.9 ± 8.8	28.6 ± 6.6	N	N	Plasma CoQ 10 concentrations
Barry 2001	4 weeks	Healthy subjects	24 (12/12)	26 ± 5	26 ± 5	41.7	41.7	Plasma CoQ 10 concentrations
Ashton et al. 2011	26 weeks	Heart failure (NYHA II or III) with LVEF < 40%	69 (32/37)	N	N	N	N	Plasma CoQ 10 concentrations

*NYHA* New York Heart Association, *LVEF* left ventricular ejection fraction, *HIV* human immunodeficiency virus, *STEMI* st segment elevation myocardial infarction, *CAD* coronary artery disease, *CK* creatine kinase, *CoQ10* coenzyme Q 10, *T* treatment group, *C* control group, *N* not mentioned

### Quality assessment

“Low risk,” “high risk,” or “unclear risk” was categorized for all 12 included studies according to 7 risk biases presented in sequence generation, allocation sequence concealment, blinding of participants and personnel, blinding of outcome assessment, incomplete outcome data, selective outcome reporting, and other potential sources of bias (Additional file 2: Figure S1) [16]. No obvious attrition bias or reporting bias was observed. Additionally, the randomization and blinding in the included articles were considered adequate in the present study according to the Cochrane Handbook [16].

### The effect of statin treatment on circulating CoQ10

**Fig. 2:**
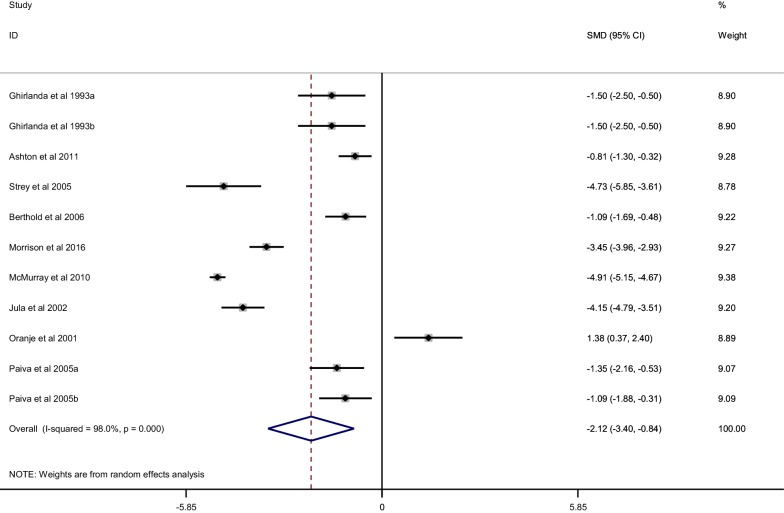
Forest plot for circulating CoQ10, statin vs placebo. *CoQ10* coenzyme Q10, *SMD* standard mean difference, *CI* confidence interval, *ID* identity number

**Fig. 3:**
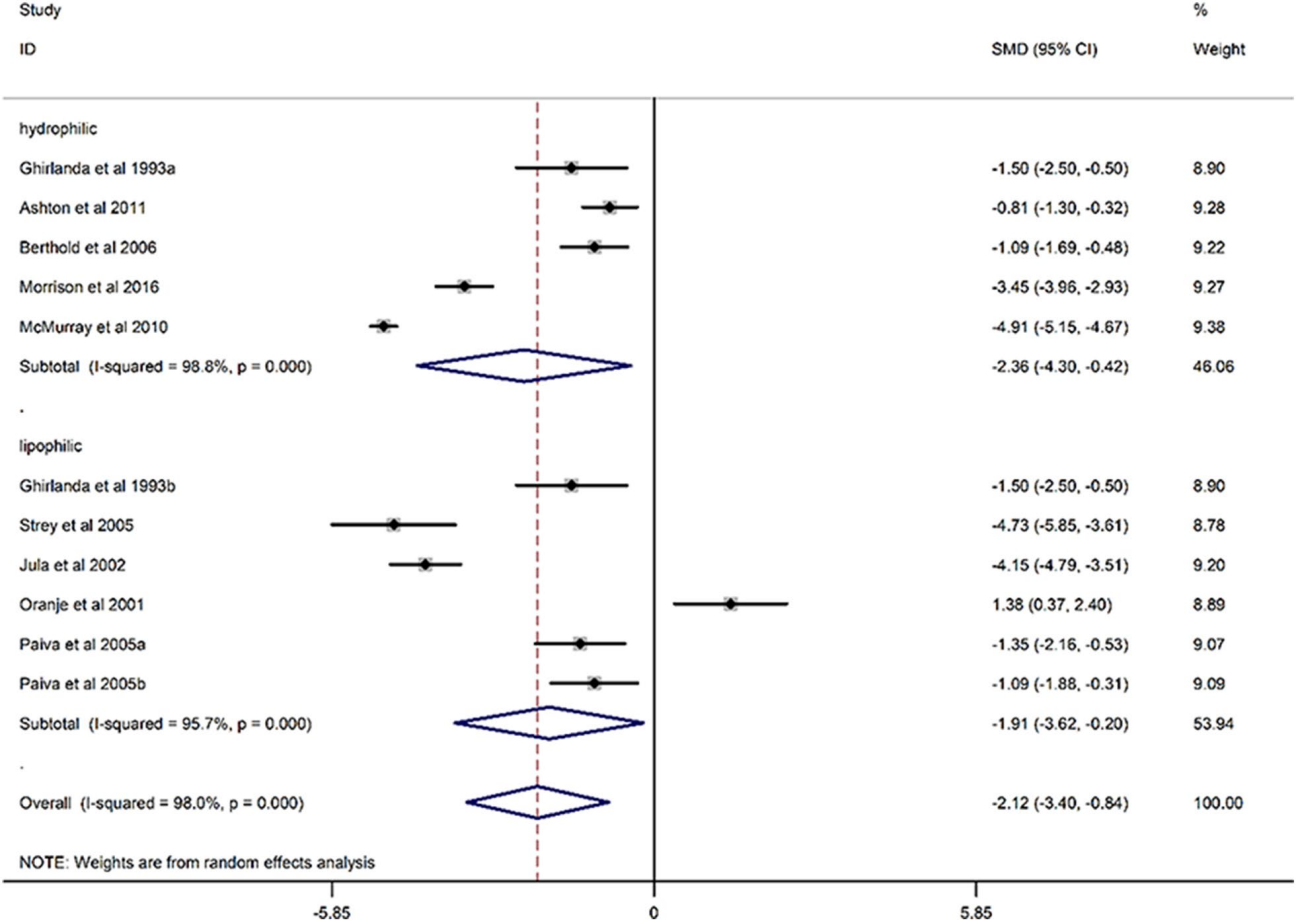
Forest plot for circulating *CoQ10* lipophilic statin vs hydrophilic statin (subgroup analysis), *CoQ10* coenzyme Q10, *SMD* standard mean difference, *CI* confidence interval, *ID* identity number

**Fig. 4:**
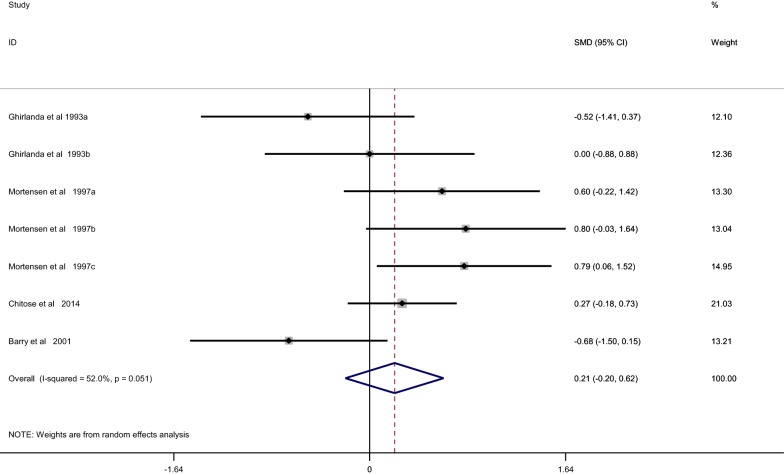
Forest plot for circulating CoQ10, lipophilic statin vs hydrophilic statin. *CoQ10* coenzyme Q10, *SMD* standard mean difference, *CI* confidence interval, *ID* identity number

**Fig. 5:**
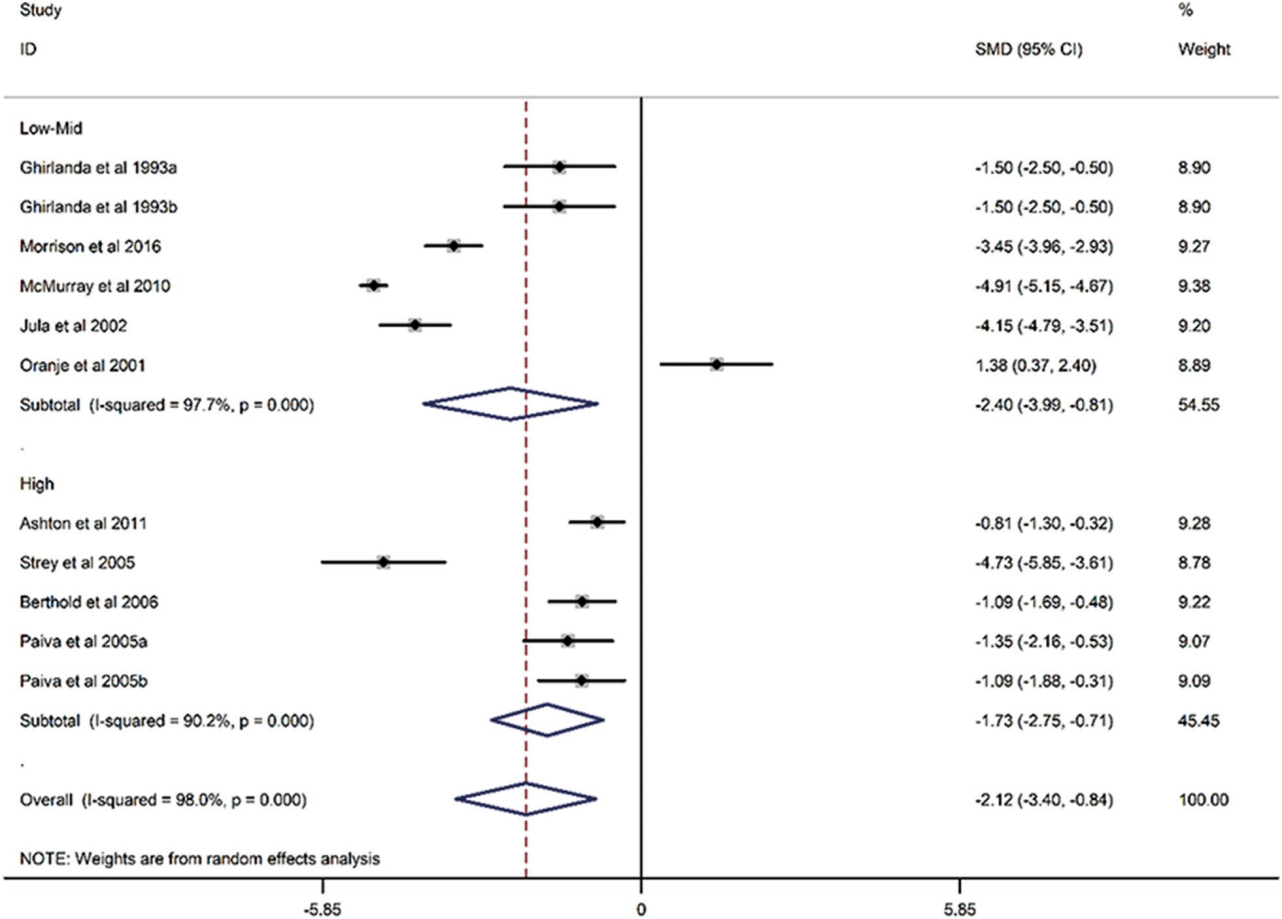
Forest plot for circulating CoQ10, low-mid intensity statin vs high intensity statin (subgroup analysis). *CoQ10* coenzyme Q10, *SMD* standard mean difference, *CI* confidence interval, *ID* identity number

**Fig. 6:**
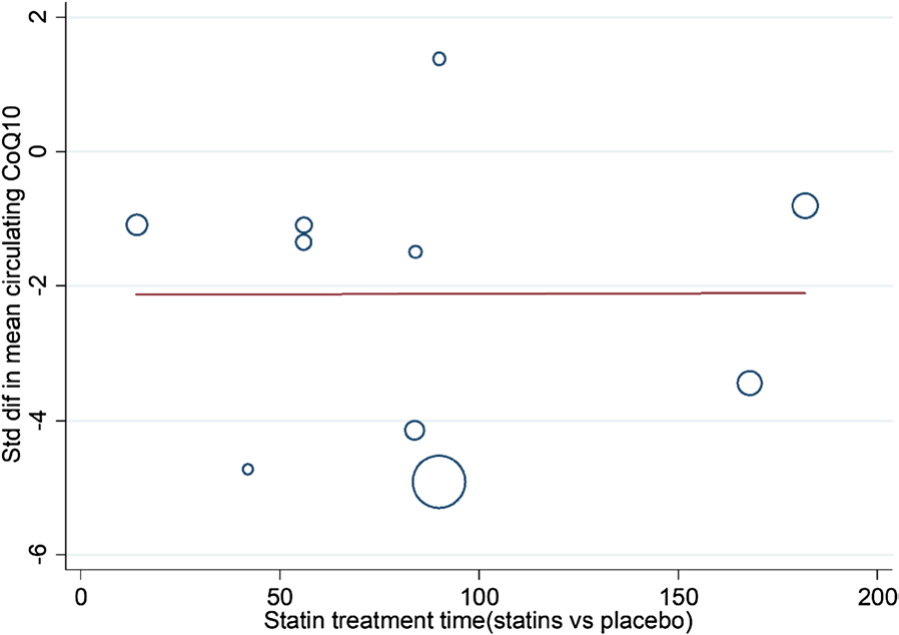
Meta-regression plot, mean change in circulating CoQ10 according to statin treatment time. *CoQ10* coenzyme Q10

When compared with placebo, statin treatment decreased circulating CoQ10 (SMD, − 2.12; 95% CI, − 3.40 to − 0.84; *p* = 0.001) with a significant heterogeneity (*I*^2^ = 98%, *p* < 0.001) (Fig. 2). The subgroup analysis demonstrated that statins could decrease circulating CoQ10 with both lipophilic statins (SMD, − 1.91; 95% CI, − 3.62 to 0.2; *p* = 0.017) and hydrophilic statins (SMD, − 2.36; 95% CI, − 4.30 to − 0.42; *p* = 0.028) (Fig. 3), and no obvious difference was observed between hydrophilic statins and lipophilic statins in the efficacy of decreasing circulating CoQ10 (SMD, − 0.20; 95% CI, − 0.208 to 0.618; *p* = 0.320) (Fig. 4). In addition, both low-middle intensity statins (SMD, − 2.403; 95% CI, − 3.992 to − 0.813; *p* < 0.001) and high intensity statins (SMD, − 1.727; 95% CI, − 2.746 to − 0.709; *p* < 0.001) could decrease circulating CoQ10 (Fig. 5). The meta-regression showed that the effect of statins on decreasing circulating CoQ10 was not closely associated with the statin treatment time (Exp, 1.00; 95% CI, 0.97 to 1.03; *p* = 0.994) (Fig. 6).

### Sensitivity analysis

To ensure the reliability of the present meta-analysis, we performed sensitivity analysis to evaluate the robustness of the pooled SMDs by eliminating each study one at a time sequentially, which indicated that the heterogeneity among the studies did not significantly change regarding the effect of statins on circulating CoQ10. Thus, no one study showed a significant impact on the results of the present meta-analysis.

### Publication bias

Three methods, including funnel plot, Egger regression test and the Begg–Mazumdar correlation test, were used to evaluate publication bias regarding the effect of statin treatment on circulating CoQ10, which suggested potential publication bias (Begg–Mazumdar correlation test, Kendall’s score = 0, continuity corrected *z* = 0.08, continuity corrected *p* = 1; Egger regression test, Coef., 9.81; 95% CI, 2.52 to 0.17.11; *p* = 0.014) (Additional file 3: Figure S2). Using a “trim and fill” correction, 5 potentially missing studies were imputed leading to a corrected effect size (SMD, − 4.01, 95% CI, − 5.38 to − 2.63, *p* < 0.0001), which was consistent with the previous effect size.

## Discussion

In the previous meta-analysis performed by Banach et al. [14], only 6 studies were included. Additionally, subgroup analysis based on the intensity of statins and comparisons between lipophilic statins and hydrophilic statins were not performed due to the limited number of enrolled studies and their small sample sizes. In the present meta-analysis, the results from 12 RCTs validated a reduction of circulating CoQ10 following statin treatment. We also showed, for the first time, that both lipophilic statins and hydrophilic statins could decrease circulating CoQ10, and there was no significant difference between the two groups. In addition, a significant effect was observed for both low-middle intensity statins and high intensity statins in terms of decreasing the level of circulating CoQ10, and the effect of statins on circulating CoQ10 was not closely associated with the statin treatment time (from 14 days to 26 weeks).

The present meta-analysis demonstrated that statin treatment decreased the level of circulating CoQ10, which is consistent with some previous clinical studies [23, 24]. The mechanisms of the reduction of circulating CoQ10 following statin treatment remain unclear though some hypotheses have been offered. First, statin treatment may decrease the biosynthesis of CoQ10. Farnesyl pyrophosphate, a precursor in the synthesis of CoQ10, was blocked during statin treatment, which might contribute to a reduction of circulating/intramuscular CoQ10 [13]. Second, statin treatment may decrease absorption of dietary CoQ10. A recent study demonstrated that statin treatment could cause gut dysbiosis in mice through activating the PXR-dependent pathway, which might influence the absorption of CoQ10 in the gut [29]. CoQ10 participates in electron transport during oxidative phosphorylation in mitochondria, protects against oxidative stress produced by free radicals, and regenerates active forms of the antioxidants ascorbic acid and tocopherol, which play important roles in maintaining mitochondrial energy metabolism and stabilizing muscle cell membranes [30–34]. CoQ10 deficiency presents as increased oxidative stress, increased inflammatory responses, and an imbalanced serotonergic system, which may contribute to SAMS [35]. The present meta-analysis validated the effect of statin in decreasing circulating CoQ10, which will be helpful for studying the mechanism of SAMS and may provide a complementary approach for SAMS treatment.

Previous studies have suggested that hydrophilic statins may have a lower rate of SAMS compared with lipophilic statins and that clinicians should consider switching to a hydrophilic statin to manage SAMS [36, 37]. However, in the present meta-analysis, no obvious difference was observed between lipophilic statins and hydrophilic statins in decreasing circulating CoQ10. Shi et al. also found that patients with SAMS who are intolerant to some hydrophilic statins may be successfully managed with simvastatin (lipophilic statin) monotherapy [38]. Therefore, whether hydrophilic statins have a lower rate of SAMS compared with lipophilic statins deserves further study. In addition, a lower dose of statin is always recommended to manage patients who tolerate statins because of SAMS [9]. However, significant effects of statins were observed in both low-moderate intensity statins and high intensity statins by decreasing circulating CoQ10 in the present study. Therefore, performing large-scale trials is necessary to compare the rate of SAMS between low-moderate intensity statins and high intensity statins. Several limitations in the present study should be noted. First, the eligible studies were heterogeneous because of certain factors, such as population characteristics, study design, and duration of statin treatment. Thus, we performed subgroup analysis, sensitivity analysis, and meta-regression to minimize the effect of heterogeneity on estimated effect size and assure the reliability of the outcomes. Second, there was potential publication bias for the effect of statin treatment on circulating CoQ10, so we used the trim and fill method to assure the robustness of the pooled results.

In conclusion, statin treatment decreases circulating CoQ10, regardless of statin solution, intensity, or treatment time. The findings provide a potential mechanism for SAMS and suggest that CoQ10 supplementation might be a promising complementary approach for SAMS.

## Additional files

**Additional file 1: Table S1.** PRISMA (Preferred Reporting Items for Systematic Reviews and Meta-Analyses) checklist [15].

**Additional file 2: Figure S1.** Risk of bias.

**Additional file 3: Figure S2.** Publication bias.
