# Supplementary material for: The effect of statin treatment on circulating coenzyme Q10 concentrations: an updated meta-analysis of randomized controlled trials
Source: Eur J Med Res. 2018 Nov 10;23:57. doi: 10.1186/s40001-018-0353-6 (PMC6230224; doi:10.1186/s40001-018-0353-6)
Supplement: Supplementary file 2 — Additional file 2: Figure S1. Risk of bias. [file 40001_2018_353_MOESM2_ESM.docx]

**Additional file**

**Additional file 2: Figure S1** Risk of bias.


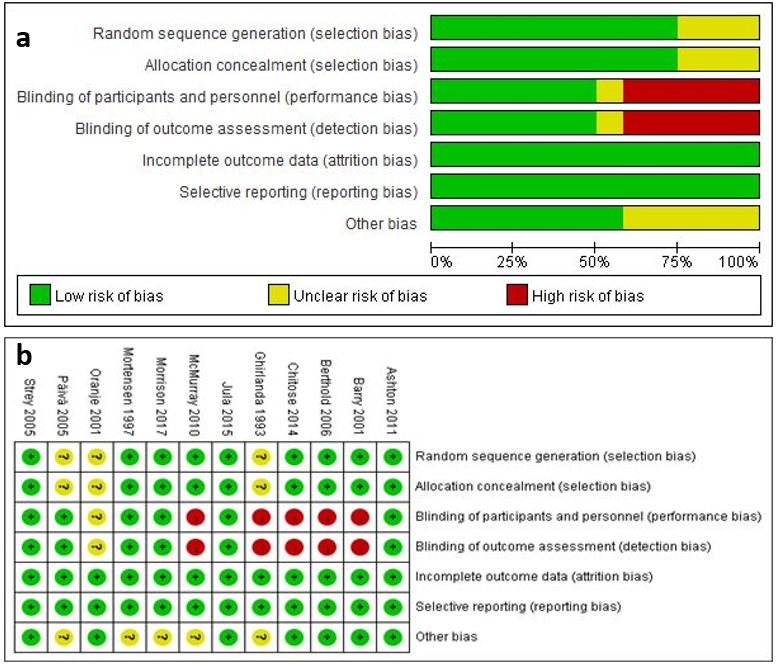


**Figure S1** Risk bias of included studies, a, risk of bias graph; b, risk of bias summary.
