# Supplementary material for: The effect of statin treatment on circulating coenzyme Q10 concentrations: an updated meta-analysis of randomized controlled trials
Source: Eur J Med Res. 2018 Nov 10;23:57. doi: 10.1186/s40001-018-0353-6 (PMC6230224; doi:10.1186/s40001-018-0353-6)
Supplement: Supplementary file 3 — Additional file 3: Figure S2. Publication bias. [file 40001_2018_353_MOESM3_ESM.docx]

**Additional file**

**Additional file 3: Figure S2** Publication bias.


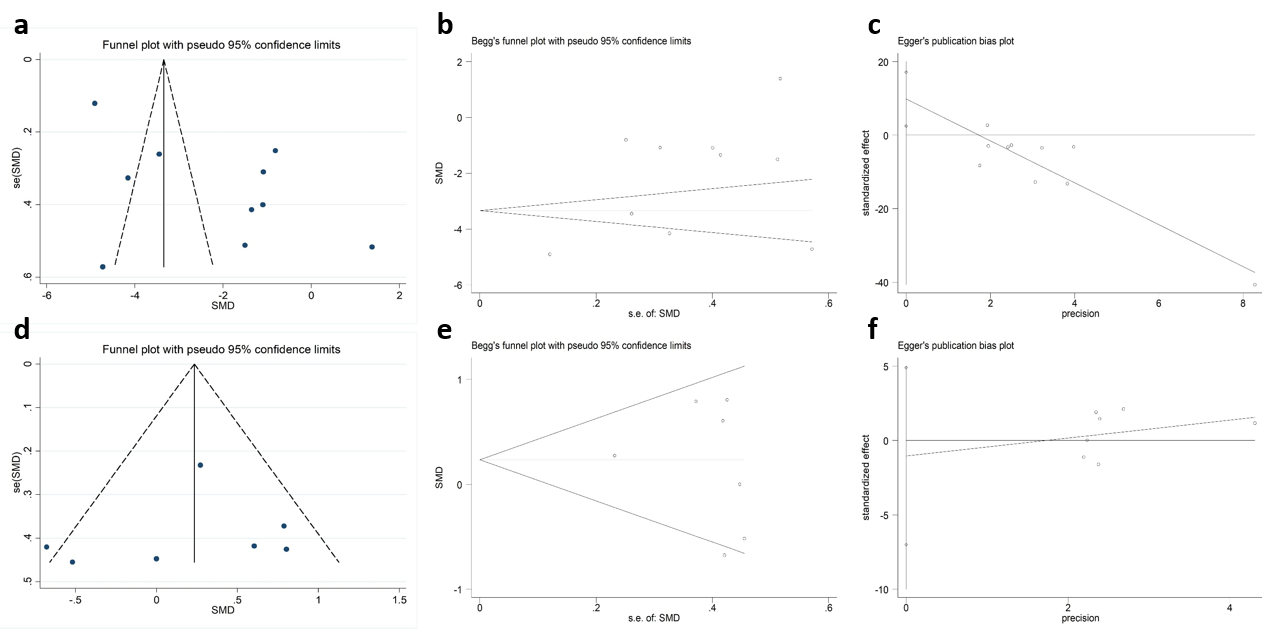


**Figure S2** Funnel plot, Begg test and Egger test for publication bias.
